# Gene Expression and Alternative Splicing Analysis in a Large-Scale Multiple Sclerosis Study

**DOI:** 10.3390/ijms252211957

**Published:** 2024-11-07

**Authors:** Müge Sak, Julia H. Chariker, Juw Won Park, Eric Christian Rouchka

**Affiliations:** 1 Kentucky IDeA Networks of Biomedical Research Excellence Bioinformatics Core, Department of Neuroscience Training, University of Louisville, Louisville, KY 40292, USA; muge.sak@louisville.edu (M.S.); julia.chariker@louisville.edu (J.H.C.); 2 Brown Cancer Center Bioinformatics Core, Center for Integrative Environmental Health Sciences Biostatistics and Informatics Facility Core, Department of Medicine, University of Louisville, Louisville, KY 40292, USA; juw.park@louisville.edu; 3 Kentucky IDeA Networks of Biomedical Research Excellence Bioinformatics Core, Department of Biochemistry and Molecular Genetics, University of Louisville, Louisville, KY 40292, USA

**Keywords:** multiple sclerosis, RNA-seq, differential expression, alternative splicing

## Abstract

Multiple Sclerosis (MS) is an autoimmune neurodegenerative disease affecting approximately 3 million people globally. Despite rigorous research on MS, aspects of its development and progression remain unclear. We utilized a publicly available RNA-seq dataset (GSE138614) consisting of the post-mortem white matter tissues of five donors without any neurological disorders and ten MS patient donors. We investigated gene expression levels correlated with tissue inflammation and alternative splicing to identify possible pathological isoforms in MS tissues. We identified RNA-binding motifs, differentially expressed RNA-binding proteins, and single-nucleotide polymorphisms (SNPs) to unravel possible mechanisms of alternative splicing. Genes with expression changes that were positively correlated with tissue inflammation were enriched in the immune system and receptor interaction pathways. Genes showing a negative correlation were enriched in nervous system development and in metabolic pathways. A comparison of normal-appearing white matter (NAWM) and active or chronic active lesions within the same donors identified genes playing roles in immunity, white matter injury repair, and remyelination. We identified exon skipping events and spontaneous SNPs in membrane-associated ring-CH-type finger-1 (*MARCHF1*), UDP glycosyltransferase-8 (*UGT8*), and other genes important in autoimmunity and neurodegeneration. Overall, we identified unique genes, pathways, and novel splicing events that can be further investigated as potential novel drug targets for MS treatment.

## 1. Introduction

Multiple Sclerosis (MS) is an autoimmune neurodegenerative disease of the central nervous system (CNS) that affects about 3 million people worldwide [1]. Magnetic resonance imaging of the brain and spinal cord identifies characteristic lesions, and evidence of intrathecally produced immunoglobulin G (IgG) via lumbar puncture is used as a diagnostic test for MS [2]. Vitamin D deficiency and Epstein–Barr virus infections have been consistently linked to MS diagnosis [3]. Although MS is not an inherited disease, genetic susceptibility to MS is a known phenomenon [3]. This includes knowledge of 233 risk loci identified in genome-wide association studies. Thirty of these loci are on the major histocompatibility complex (MHC), suggesting a primary role of autoimmunity in MS [4].

MS is a complex disease with an unclear and multifactorial origin and lesion pathology. Lesions are characterized by inflammation, demyelination, and neurodegeneration of the CNS. These lesions differ in distribution and composition both among different patients and within individuals [5]. Their specific locations often correlate with clinical symptoms, leading to significant clinical variability [6,7]. Several biological processes take place during the progression of MS: autoreactive T-cell and B-cell aggregation in the CNS, the secretion of cytokines, and damage to the myelin sheath and to oligodendrocytes [8]. The timing of these processes resulting in neurodegeneration is important for therapeutic strategies [9]. Various imaging techniques have tested variables relevant to the timing of disease processes, leading to the development of staging systems for MS [6,10]. However, there is no standard method for the classification of lesions. The histological staging system for post-mortem tissues includes staining inflammatory cells, glial cells, axonal loss, and myelin [10,11].

Here, we utilized publicly available RNA-seq data from the post-mortem white matter (WM) tissues of five non-MS and ten MS patient donors [12] (Table S1). A previous analysis with this dataset identified lesion-specific molecular signatures, protein complex networks, and the transforming growth factor beta receptor 2 (TGFβ-R2) as a central hub [13]. To extract additional information from this dataset, we employed differential expression, differential splicing, and variant analysis both among and within donors. This approach identified genes that may play a role in the progression of inflammation. Through non-MS vs. MS tissue comparisons, we identified over 6000 differentially expressed genes (false discovery rate (FDR) < 0.05). The most differentially expressed genes were involved in inflammation, immune system processes, and cell signaling. In comparing WM to different lesion types, we investigated genes that follow the same patterns with relation to inflammation level, from normal-appearing white matter (NAWM) to active lesions (AL) in the tissues. The genes that show a positive correlation with inflammation were enriched in immune system processes, in immune response and response to stimulus, and in KEGG pathways such as cytokine–receptor interaction. Negatively correlated genes were enriched in neurogenesis and nervous system development in biological processes and pathways. Donor-specific differential expression analysis showed that immunoglobulin gene subfamilies were differentially expressed. Finally, differential splicing and variant analyses identified genes that play a role in dysregulation in the immune system, metabolism, and axon/dendrite structure stability.

Currently, there is no cure for MS. Disease-modifying therapies help with recovery from attacks, modify the course of the disease, and manage symptoms. Our comprehensive analysis of differential expression and the splicing of genes in MS patients demonstrates the role of previously recognized genes, as well as novel pathways. Our results can lead the way in identifying novel genes and drug targets for reversing damage, preventing MS progression, and helping patients with disease management.

## 2. Results

### 2.1. Differentially Expressed Genes in Non-MS Versus MS Samples

**Figure 1:**
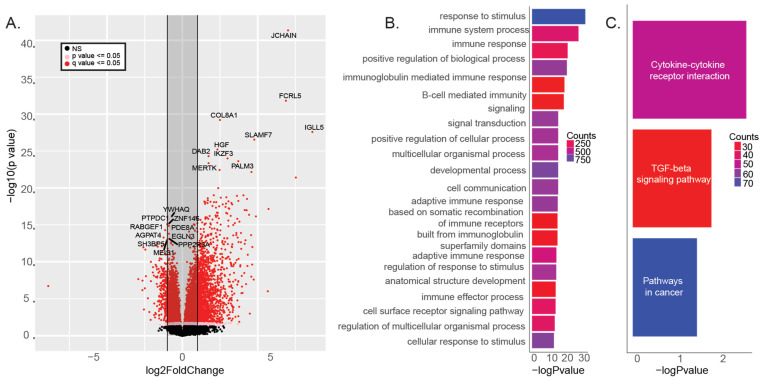
Differential expression of WM tissues from MS and non-MS donors. (**A**) Volcano plot showing differentially expressed genes between MS and non-MS donor tissue samples. (**B**) Enriched gene ontology biological processes (GO:BP) and (**C**) KEGG pathways from the differentially expressed genes.

We found 6,461 differentially expressed genes between MS and non-MS tissue samples (FDR < 0.05) (Figure 1A). Among these, the joining chain of multimeric IgA and IgM (*JCHAIN/IGJ*) was identified as the most significant differentially expressed gene in white matter lesions from MS donors. Other genes with high expression in MS patient samples were proinflammatory, such as immunoglobulin lambda-like polypeptide 5 (*IGLL5*), collagen type VIII alpha 1 chain (*COL8A1*), signaling lymphocytic activation molecule family member 7 (*SLAM7*), paralemmin 3 (*PALM3*), and hepatocyte growth factor (*HGF*). Additionally, overexpressed genes such as IKAROS family zinc finger 3 (*IKZF3*), MER proto-oncogene, tyrosine kinase (*MERTK*), and Fc receptor-like 5 (*FCRL5*) all have risk alleles associated with MS or other autoimmune diseases [14,15] (Figure 1A). The most significantly downregulated genes were primarily involved in signal transduction and biosynthesis pathways, including tyrosine 3-monooxygenase/tryptophan 5-monooxygenase activation protein theta (*YWHAQ*), protein tyrosine phosphatase domain containing 1 (*PTPDC1*), RAB guanine nucleotide exchange factor 1 (*RABGEF1*), 1-acylglycerol-3-phosphate O-acyltransferase 4 (*AGPAT4*), and SH3 domain binding protein 5 (*SH3BP5*) (Figure 1A). Functional enrichment analysis revealed that these differentially expressed genes were significantly involved in stimulus response, the immune system, and cell signaling functions (Figure 1B), as well as in proinflammatory cytokine–cytokine receptor interaction and TGF-beta signaling pathways (Figure 1C).

### 2.2. Genes Showing Expression Positively or Negatively Correlated with Tissue Inflammation

**Figure 2:**
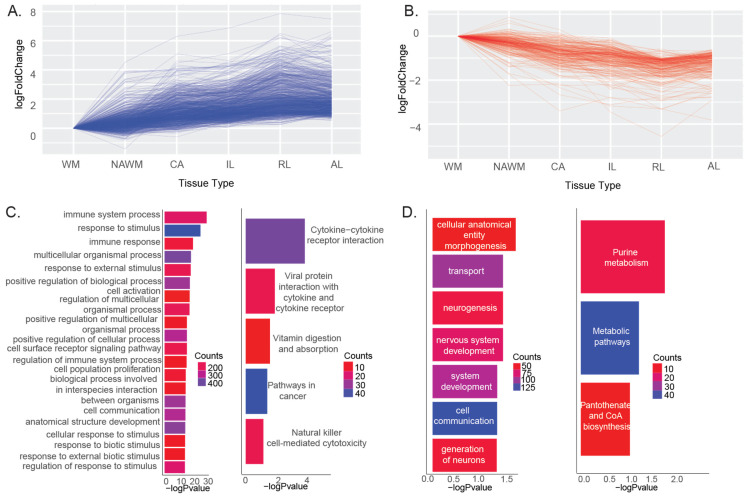
Genes showing expression changes that are (**A**) positively and (**B**) negatively correlated with tissue inflammation levels. (**C**) Enriched GO:BP and KEGG pathways for genes showing expression levels positively correlated with inflammation. (**D**) Enriched GO:BP and KEGG pathways for genes showing expression levels negatively correlated with inflammation.

We identified 2886 genes which have a positive correlation with the inflammation levels in the tissues (Figure 2A) and 647 genes with a negative correlation (Figure 2B). Positively correlated genes enriched in biological pathways were similarly identified through the differential expression analysis, such as in immune system process, immune response, and response to stimulus (Figure 2C). Additionally, we identified viral protein interaction with cytokine receptors, vitamin digestion and absorption, and natural killer cell-mediated cytotoxicity pathways associated with inflammation levels in MS tissues. Genes showing expression levels negatively correlated with tissue inflammation were enriched in the cellular anatomical entity morphogenesis, neurogenesis, and metabolic pathways (Figure 2D).

### 2.3. Differential Splicing and Differential Expression of RNA-Binding Proteins (RBPs)

**Figure 3:**
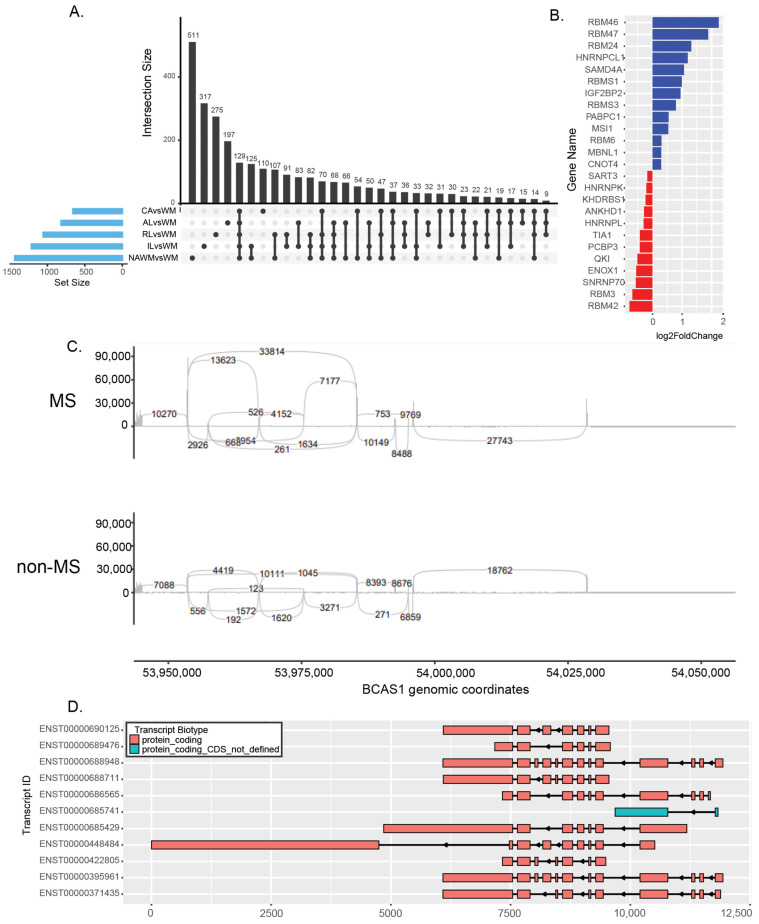
Differentially spliced genes in all tissues from MS donor samples compared to non-MS samples. (**A**) UpSet graph showing the number of alternatively spliced genes in each category of tissues and intersections. (**B**) Differentially expressed RNA-binding proteins involved in alternative splicing. (**C**) Sashimi plot of the differentially spliced *BCAS1* gene in MS and non-MS tissues. The numbers represent the sequence-based junction counts. (**D**) Exon structure of known *BCAS1* transcripts.

We identified differentially spliced genes in each tissue that was compared to non-MS WM, with a total of 2721 genes alternatively spliced in MS tissues, and 1389 of these genes were not differentially expressed. The intersections of alternative splicing events across comparisons are illustrated in Figure 3A. The representative motif bindings for each type of splicing are shown in Figure S1, and differentially expressed RNA-binding proteins (RBPs) that bind to these motifs in MS lesions are shown in Figure 3B. These suggest that the mechanism of alternative splicing may result from the differential expression of RBPs. Key genes with alternative splicing included myelin basic protein (*MBP*), DEAD-box helicase 5 (*DDX5*), KH domain-containing RNA-binding protein (*QKI*), and discs large MAGUK scaffold protein 1 (*DLG1*) (Table 1). We also found that MS patients had significant exon skipping events in brain-enriched myelin-associated protein (*BCAS1*) that can result in the expression of alternative isoforms (Figure 3C,D).

### 2.4. Comparison of NAWM with AL or CA Lesions Within Donors

**Figure 4:**
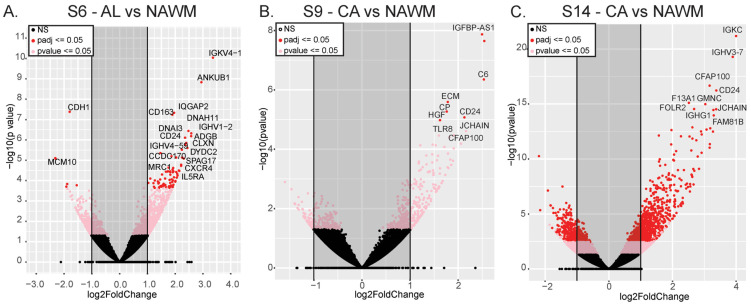
Volcano plots of differentially expressed genes in different tissue types from the same donors. (**A**) AL compared to NAWM in donor S6; (**B**) CA lesions compared to NAWM in donor S9; and (**C**) S14 CA vs. NAWM.

We examined differential expression and splicing within the same donors to minimize inter-donor variation and closely analyzed the CA and AL lesions by comparing them to the NAWM from the same donor. This approach enabled a detailed and specific examination of the molecular differences in the lesion areas of patient brains. A comparison of NAWM and AL showed increased expression of *IGKV4-1*, *IGHV1-2*, and *IGHV4-59* as well as local inflammation marker *CD163* and interleukin-5 receptor alpha (*IL5RA*) (Figure 4A, Table S2). *CD24*, which carries polymorphisms linked to the progression of autoimmune disorders [16], and *JCHAIN* genes were differentially expressed in both AL and CA lesions compared to NAWM for three donors. The *IGHV3-30* gene was overexpressed in the CA and NAWM lesion comparison for donor S9 (Figure 4B, Table S3). For donor S14, we found that the immunoglobulin genes *IGKC, IGHV3-7, IGHG1, IGHV4-39*, and *IGHG3* were overexpressed in CA lesions compared to NAWM (Figure 4C, Table S4). In addition to *CD24*, the CA and NAWM lesion comparison for donors S9 and S14 both showed overexpression of hepatocyte growth factor (*HGF*), extracellular matrix protein (*ECM2*), complement 6 (*C6*), ceruloplasmin (*CP*), and insulin growth factor-binding protein 7 antisense RNA 1 (*IGFBP7-AS1*) (Figure 4B,C, Tables S3 and S4). This suggests these genes may have specific roles in CA lesions.

Differentially expressed AL genes were enriched in cilium movement, mainly through the upregulation of cilia- and flagella-associated protein 100 (*CFAP100)*, cilia- and flagella-associated protein 45 (*CFA45*), and dynein axonemal heavy chain 5 (*DNAH5*). Negative enrichment of postsynaptic membrane potential regulation indicates substantial damage to neurons in which synaptic failure eventually leads to brain network alterations and contributes to disabling MS symptoms and disease progression [17] (Table S5). Splice variants in AL compared to NAWM lesions were mainly enriched in metabolic processes. We found significant disruption of cellular localization by alternatively spliced genes (Supplemental Figure S2A) that could be involved in disrupted interleukin receptor localization in MS tissues [18].

Gene set enrichment analysis for differentially expressed genes in CA lesions and NAWM showed that B-cell-mediated immunity and axoneme assembly were the most positively enriched pathways. Negatively enriched short-chain fatty acid and acetyl-COA metabolic processes have been shown to have immunomodulatory potential in MS [19] (Tables S6 and S7). Differentially spliced genes in the CA lesions compared to the NAWM from the same patients were mostly enriched in cellular component organization, nervous system development, and cell morphogenesis pathways, indicating possible pathogenicity due to the contribution of alternative splicing to mitochondrial and metabolic dysfunction of the CA lesions (Figure S2B).

### 2.5. Spontaneous Single-Nucleotide Variations and Alternative Splicing

A comparison of the single-nucleotide variations (SNVs) in MS and non-MS samples did not result in matches of any known SNV markers of MS, likely due to the small number of donors. We then compared SNVs in AL and CA lesions to NAWM lesions in donors S6, S9, and S14, and identified a common synonymous variation (rs4947) in heat shock protein 90 alpha family class A member 1 (*HSP90AA1*) across all comparisons (Table 2). We also identified SNVs on Rho GTPase activating protein 21 (*ARHGAP21*), contactin 1 (*CNTN1*), solute carrier family 1 member 2 (*SLC1A2*), UDP glycosyltransferase 8 (*UGT8*), solute carrier organic anion transporter family member 1C1 (*SLCO1C1*), protocadherin 9 (*PCDH9*), and membrane-associated ring-CH-type finger 1 (*MARCHF1*) (Table 2). In comparing CA lesions to NAWM tissues, *ARHGAP21* had significant exon skipping events in donors S9 and S14, as well as in the AL lesions of donor S6. *SLC1A2* and *CNTN1* genes had significant SE events in S14. *CNTN1* also showed an SE variant in donor S6’s AL lesions. The same events were found for *SLC1A2* and *CNTN1* genes in S9 but were not significant. *PCDH9* carried spontaneous SNVs in all comparisons and had significant SE events in both the AL and CA lesions from donors S6 and S9 when compared to their NAWMs (Table 2).

**Figure 5:**
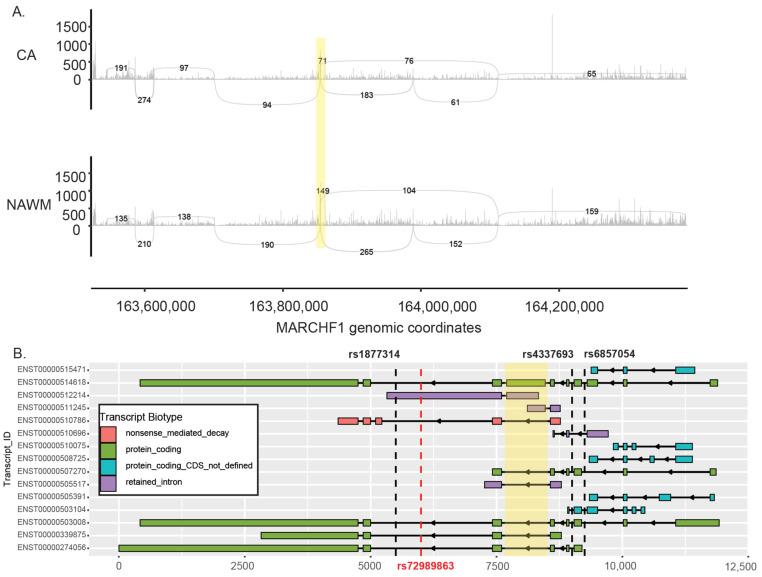
(**A**) Sashimi plot of the *MARCHF1* gene that is differentially spliced in CA lesions. The numbers represent the sequence-based junction counts. (**B**) Exon structure of known transcripts of *MARCHF1*. The known MS risk allele is shown in red (rs72989863), and the identified spontaneous SNPs are shown in black. The skipped exon 4 is highlighted in yellow in both (**A**,**B**).

MARCHF1 is an E3 ubiquitin ligase that mediates the ubiquitination of MHC II, which impacts the MHC I antigen presentation pathway [20]. It carries a known MS risk variant rs72989863. *MARCHF1* was not differentially expressed in any donor-specific comparisons. We identified different spontaneous SNVs that are genic upstream variants in CA lesions from both donors S9 and S14. Donor S14 also had a significant SE variant in their CA lesions (Table 2, Figure 5A). The same region was also skipped in the CA lesions from donor S9 but were not significant. In donor S6, the comparison of AL and NAWM tissues found the same SE event on *MARCHF1*. This region corresponds to exon 4 of the ENST00000514618 transcript, and this exon skipping event may result in the expression of the ENST0000503008 transcript as it is the only other full-length protein coding transcript without exon 4 (Figure 5B). These two transcripts translate into two different MARCHF1 protein isoforms that may alter E3 ubiquitin ligase activity (uniport IDs Q8TCQ1 and D6RGC4). However, it is essential to note that our analysis utilized short-read sequencing data, which does not allow the identification of specific transcripts.

## 3. Discussion

MS is highly complex and variable, both among patients and among the stages of the disease. Understanding the molecular processes through disease progression and identifying molecular signatures of the specific pathology of MS lesions are crucial for understanding the disease’s development and progression, as well as for patient stratification for prognosis, predicting treatment response, and identifying treatment strategies. The dataset we utilized has a large number of samples from different types of MS lesions, which allowed us to investigate the differences in these lesions with different approaches. Clustering these lesions according to their molecular characteristics was challenging, likely due to the dynamic inflammatory activities and repair mechanisms occurring during the evolution of individual lesions [21]. Identifying gene expression patterns correlating with tissue inflammation provides insights for the identification of lesion stages.

Alternative splicing is one of the main mechanisms affecting the expression of genes and gene isotypes. A recent study showed there are a large number of alternative splicing variants in MS that are not linked to differential expression [22], and there are splice variants that have been found to be associated with known risk alleles [23]. Therefore, differential expression alone does not provide sufficient information, as splicing offers additional insights. Many of the alternatively spliced genes we identified play roles in MS-related pathways.

Deletion of *QKI* isoforms in oligodendrocytes leads to severe CNS hypomyelination accompanied by tremors [24]. DLG1 plays a crucial role in lymphocyte activation and has known isoforms that regulate p38-dependent and independent effector functions in CD8+ T cells [25]. *BCAS1* is highly expressed in oligodendrocytes and plays a role in demyelination in MS [26]. We discovered that *BCAS1*, which carries a known MS risk allele rs2585447 [4], had a splice variant in MS patient samples. Our analysis also identified differential splicing of *MBP* and *MOBP* in different types of MS lesions that show variable expression levels in MS [27,28]. Studying these alternative isoforms more thoroughly could enhance our comprehension of how MS progresses and how we might be able to halt or reverse it.

RBPs regulate the expression of genes, from transcription and splicing to translation. Deregulated RNA-binding protein expression may cause several different pathophysiological effects, such as misfolding, aggregation, and changes in the splicing and stability of RNAs [29]. Dysfunctional RBPs have been shown to contribute to neurodegeneration. Specifically, heterogeneous nuclear ribonuclear protein A1 (*hnRNP A1*) dysfunction was shown in neurons in the brains of MS patients [30]. Our results identify significantly enriched RNA-binding motifs that were involved in alternative splicing events and highlight differentially expressed RNA-binding proteins as a potential mechanism for alternative splicing.

Although we had limited samples for the tissue type comparisons within donors, our approach to investigating the differential expression and differential splicing of CA lesions vs. NAWM lesions and AL vs. NAWM lesions resulted in the identification of specific pathways such as fatty acid and acetyl-COA metabolic processes, as well as cellular components and cytoskeleton organization. Lipids are not solely involved in the formation of the myelin sheath but are found to be important components of cell signaling, communication, and transport in the CNS, and have been shown to be relevant players in neuroinflammation and neurodegeneration [31]. These specific pathways affected in specific stages of MS lesions can be markers for the staging of these lesions, as well as for the selection of treatment strategies. The identified pathways are also a basis of further research in reversing damage and treating disability symptoms of MS. The inflammatory activities in lesions are shown to result in disease progression without any new lesion formation [32]. In AL, we identified markers for neuronal damage by changes in cilia and postsynaptic membrane potential pathways. Targeting these specific proteins and pathways may block the silent progression of disease and prevent further disability development in patients.

A comparison of NAWM tissues with CA lesions or AL within patients identified spontaneous SNVs that may be associated with alternative splicing in specific MS lesions. Among the genes we identified, *ARHGAP21*, *SLC1A2*, and *CNTN1* are involved in signaling for synaptic and immune homeostasis, and in axon/dendritic transport regulation [33,34,35,36]. Also, UGT8 plays an important role in remyelination by mediating the major myelin lipid galactosylceramide [37]. Alternative splicing of these genes may result in dysregulation of neuron activity and myelination. MARCHF1 mediates the surface turnover of MHC class II and regulates type I interferon signaling, T-cell activation, and IFN-γ production during infections [38]. Moreover, deletion of the 164,703,186 to 165,032,803 residues in *MARCHF1* causes growth failure, developmental and speech delays, and aggressive behavior [39]. We were able to predict isoforms of *MARCHF1* that are expressed in MS lesions and NAWM. Identification of the isoforms is important to understand the mechanisms of MS, prevent MS progression, and reverse neuronal damage. However, as noted, further studies require more specific methods for the identification of protein isotypes such as IsoSeq and deep proteome sequencing [40,41].

Our analysis, expectedly, showed the involvement of B-cell-mediated immunity in MS lesions. We found brain-derived neurotrophic factor (*BDNF*) to be one of the differentially spliced genes that is secreted by B cells, which prevents axonal loss and is highly expressed in the actively demyelinating area [42]. Recent studies show that IgG constant region polymorphisms affect antibody stability and dynamics, and in an MS preferential pairing of the *IGHV4* with the *IGKV1* gene family, the *IGHV4-39* gene is identified as the most abundant subisotype [43]. Our data showed overexpression of different Ig isotypes and subisotypes in AL and CA lesions. It is important to acknowledge that we were able to compare these lesions to NAWM within samples from only two donors for CA lesions and one donor for AL. However, our results suggest a novel approach to the comparison of different lesion stages that could identify specific immunoglobulin gene usages as well as specific biomarkers for molecular classifications.

Complementing disease-modifying therapies with a myelin repair treatment is an ideal approach for MS disease management [44]. Treatment approaches for myelin repair focus on stimulating the signaling between neurons and oligodendrocyte precursor cells for remyelination [45]. Currently, there are several clinical trials for remyelination in MS patients, including trials testing the effects of vagus nerve stimulation (NCT06641271), thyroid hormone (NCT02760056), a type 2 diabetes drug metformin (NCT05893225), and an N-methyl-D-aspartate (NMDA) receptor inhibitor ifendropil (NCT06330077). Our findings show that alternatively spliced genes significantly affect cell signaling. We also identified alternative splicing in genes involved in metabolism, myelin production, and remyelination. Our results provide a novel approach to identifying the key factors in myelin repair through investigating alternative splicing and potential new drug design strategies.

Our analysis explored various methods of and approaches to identifying novel molecular characteristics of MS lesions in the brain. We discovered that metabolic pathways were highly affected by gene expression changes. We provided additional insight to differential expression with our differential splicing analysis and identified genes with crucial roles in MS-related pathways that have alternative isotypes in MS lesions. Our findings identified RNA-binding motifs that are important in alternative splicing events. Additionally, through the comparison of lesions within donors, we found spontaneous SNVs and alternative isoforms in genes that are essential factors in autoimmunity, neuron homeostasis, and myelination that may have pivotal roles in MS lesions. Overall, our results indicate that splice variants in specific MS lesions may be used as biomarkers to determine the staging of lesions as well as treatment targets.

## 4. Materials and Methods

### 4.1. Data Access and Characteristics

The publicly available RNA-seq dataset was retrieved from the GEO repository (accession GSE138614). The original study that generated the data was approved by the local ethics committee of the original authors. The lesion classification method was explained in Elkjaer et al. [13]. Namely, NAWM, AL, chronic active (CA), inactive (IL), and remyelinating (RL) lesions were characterized by myelin integrity and the inflammatory state [10]. The accession numbers for the comparisons of NAWM tissues with AL or CA lesions within the same donors are shown in Table S1.

### 4.2. Differential Expression Analysis

Reads from 73 tissues from 10 MS donors and 25 tissues from 5 non-MS donor control samples were mapped to the human genome (hg38) using the Spliced Transcripts Alignment to a Reference (STAR) aligner (version 2.6) [46]. Raw gene counts were determined using HTSeq-count [47] (version 0.10.0). Raw counts were then normalized using the relative log expression method and filtered to exclude genes with fewer than 10 counts across all samples. Differential expression analysis was performed with DESeq2 [48] (version 1.46.0) using a negative binomial regression model to analyze pairwise comparisons. Statistical significance was determined using an FDR (q-value) cutoff of 0.05.

### 4.3. Inflammation Level Analysis

STEM is an algorithmic approach for clustering, comparing, and visualizing short-time series gene expression [49]. This clustering algorithm selects a set of distinct and representative temporal expression profiles that are independent of the data. Each gene is assigned to the model profile that most closely matches the expression profile, determined by the correlation coefficient. A permutation test determines the assignments of genes to model profiles. The significance of each model profile is calculated by the number of assigned genes under the true ordering of time points compared to the average number assigned to the model profile [49]. STEM was used to investigate the correlation between gene expression and tissue inflammation. Instead of using time series samples, the inflammation level of each sample was used to distinguish genes that showed positive and negative correlations.

### 4.4. Alternative Splicing Analysis

Replicate multivariate analysis of transcript splicing (rMATS v3.2.5) [50] was used to identify differentially spliced genes. rMATS employs a modified generalized linear mixed model to identify differential splicing from RNA-seq data with replicates. Using both splice junction and exon body read counts as input, rMATS computes the percent splicing index and the FDR for five major types of splicing events: skipped exons (SE), mutually exclusive exons (MXE), retained introns (RI), and 5′ and 3′ alternative splice sites (A5SS and A3SS). Motif enrichment was analyzed in the proximity of alternatively spliced exons using RNA Map Analysis and Plotting Server 2 (rMAPS2) [51]. rMAPS2 analyzes differential alternative splicing data obtained from rMATS and graphically displays enriched motif sites.

### 4.5. Functional Enrichment Analysis

Gene ontology biological processes (GO:BP) and Kyoto Encyclopedia of Genes and Genomes (KEGG) enrichments were determined via hypergeometric testing using cluster-Profiler [52] (version 4.14.0) for differentially expressed and differentially spliced genes in R Studio (2023.06.1). The gene set enrichment analysis tool (GSEA 4.3.2) was utilized on pre-ranked differentially expressed genes in comparisons of lesions within samples [53].

### 4.6. RNA-Seq Short Variant Discovery

RNA-seq short variants were identified using the Genome Analysis Toolkit (GATK 4.2.0.0) workflow as a guide [54]. Alignment files were preprocessed using Picard’s (v_2.25.6) AddOrReplaceGroups and GATK’s (v-4.2.0.0) tool MarkDuplicates. SplitNCigarReads formatted the RNA-seq alignments for use in HaplotypeCaller. Base quality and recalibration were performed to correct for systematic errors in base quality scores prior to using HaplotypeCaller for variant calls. SNVs were filtered based on Fisher Strand values (FS > 30.0) and Qual by Depth values (QD < 2.0).

## Figures and Tables

**Table 1:** Differentially spliced genes in the MS patient samples. (A3SS: alternative 3′ splice site; A5SS: alternative 5′ splice site; RI: retained intron; SE: skipped exon.)

Gene Symbol	Splicing Event	Gene Description
*QKI*	A3SS	KH Domain-Containing RNA-Binding Protein
*DDX5*	A5SS/RI/SE	DEAD-Box Helicase 5
*NAP1L1*	A5SS	Nucleosome Assembly Protein 1-Like 1
*EIF4G2*	RI	Eukaryotic Translation Initiation Factor 4 Gamma 2
*HMGN1*	RI/SE	High-Mobility Group Nucleosome-Binding Domain 1
*RBM39*	RI/SE	RNA-Binding Motif Protein 39
*RPL7A*	RI	Ribosomal Protein L7a
*SMARCC2*	RI	SWI/SNF-Related, Matrix-Associated, Actin-Dependent Regulator of Chromatin Subfamily C Member2
*ANLN*	SE	Anillin, Actin-Binding Protein
*ARID4B*	SE	AT-Rich Interaction Domain 4B
*BCAS1*	SE	Brain-Enriched Myelin-Associated Protein 1
*CD44*	SE	CD44 Molecule
*DLG1*	SE	Discs Large MAGUK Scaffold Protein 1
*DST*	SE	Dystonin
*EIF4A2*	SE	Eukaryotic Translation Initiation Factor 4A2
*EPB41L2*	SE	Erythrocyte Membrane Protein Band 4.1-Like 2
*ERBIN*	SE	Erb-B2 Receptor Tyrosine Kinase 2
*FGF1*	SE	Fibroblast Growth Factor 1
*FIP1L1*	SE	Factor Interacting with PAPOLA and CPSF1
*GLIS3*	SE	GLIS Family Zinc Finger 3
*HNRNPH3*	SE	Heterogeneous Nuclear Ribonucleoprotein H3
*ITSN1*	SE	Intersectin 1
*LRRC63*	SE	Leucine-Rich Repeat Containing 63
*MACF1*	SE	Microtubule Actin Crosslinking Factor 1
*MAP4*	SE	Microtubule-Associated Protein 4
*MAP4K4*	SE	Mitogen-Activated Protein Kinase Kinase Kinase Kinase 4
*MBP*	SE	Myelin Basic Protein
*NCAM1*	SE	Neural Cell Adhesion Molecule 1
*NCOA6*	SE	Nuclear Receptor Coactivator 6
*NDRG2*	SE	NDRG Family Member 2
*PICALM*	SE	Phosphatidylinositol Binding Clathrin Assembly Protein
*PRDM2*	SE	PR/SET Domain 2
*PTK2*	SE	Protein Tyrosine Kinase 2
*PTPN11*	SE	Protein Tyrosine Phosphatase Non-Receptor Type 11
*PTPRD*	SE	Protein Tyrosine Phosphatase Receptor Type D
*PXK*	SE	PX Domain Containing Serine/Threonine Kinase Like
*SHTN1*	SE	Shootin 1
*SLTM*	SE	SAFB-Like Transcription Modulator
*SORBS1*	SE	Sorbin And SH3 Domain Containing 1
*SOX2-OT*	SE	SOX2 Overlapping Transcript
*SPECC1*	SE	Sperm Antigen with Calponin Homology and Coiled-Coil Domains 1
*TLE4*	SE	TLE Family Member 4, Transcriptional Corepressor
*TMEM165*	SE	Transmembrane Protein 165
*WAC*	SE	WW Domain Containing Adaptor with Coiled-Coil
*ZNF207*	SE	Zinc Finger Protein 207
*ZNF638*	SE	Zinc Finger Protein 638

**Table 2:** Common SNPs and alternative splicing events in comparisons of AL and CA tissues to NAWM. (SE: skipped exon; RI: retained intron; MXE: mutually exclusive exons.)

CA vs. NAWM S9				
Gene Symbol	SNP Coordinate	SNP ID	Significant AS Events	AS Coordinate
*ARHGAP21*	chr10:24,621,826	rs4262623	SE	chr10:24,666,984–24,667,009
*SLCO1A2*	chr12:21,324,153	rs10459075	RI	chr12:21,293,944–21,295,792
*PCDH9*	chr13:66,988,744	rs9540962	SE	chr13:66,782,713–66,782,797
*SLC1A2*	chr11:35,259,553	rs10742339		
*CNTN1*	chr12:40,733,457	rs11178118		
*UGT8*	chr4:114,652,891	rs11931776		
*UGT8*	chr4:139,509,427	rs1354563728		
*MARCHF1*	chr4:164,241,452	rs4337693		
*MARCHF1*	chr4:163,974,090	rs6857054		
*MARCHF1*	chr4:163,851,505	rs7657544		
*HSP90AA1*	chr14:102,084,466	rs4947		
**CA vs. NAWM S14**				
**Gene Symbol**	**SNP Coordinate**	**SNP ID**	**Significant AS Events**	**AS Coordinate**
*ARHGAP21*	chr10:24,620,020	not reported	SE	chr10:24,670,217–24,670,397
*CNTN1*	chr12:40,943,633	rs1056019	SE	chr12:40,763,095–40,763,198
*HSP90AA1*	chr14:102,084,466	rs4947		
*MARCHF1*	chr4:163,568,866	rs1877314	SE	chr4:163,854,020–163,854,169
*PCDH9*	chr13:66,953,673	rs9540955		
*PCDH9*	chr13:67,216,229	rs7489531		
*SLC1A2*	chr11:35,258,109	rs10768121	SE/MXE	chr11:35,322,560–35,322,679, chr11:35,380,380–35,380,541, chr11:35,418,949–35,419,007, chr11:35,418,949–35,419,558,chr11:35,292,286–35,292,520
*SLCO1C2*	chr12:20,704,542	rs10770706		
*UGT8*	chr4:114,668,146	rs11098262		
*HSP90AA1*	chr14:102,084,466	rs4947		
**AL vs. NAWM S6**				
**Gene Symbol**	**SNP Coordinate**	**SNP ID**	**Significant AS Events**	**AS Coordinate**
*HSP90AA1*	chr14:102,084,466	rs4947		
*PCDH9*	chr13:67,216,229	rs7489531	SE	chr13:66,782,713–66,782,797
*UGT8*	chr4:114,668,146	rs11098262		
*ARHGAP21*			SE	chr10:24,590,205–24,590,453
*CNTN1*			SE	chr12:40,910,072–40,910,105

## Data Availability

The samples utilized in this analysis are publicly available in GEO accession GSE138614. The differential expression, differential splicing, and variant calling files will be provided upon reasonable request.

## Supplementary Materials

The supporting information can be downloaded at https://www.mdpi.com/article/10.3390/ijms252211957/s1.
