# Supplementary material for: Gene Expression and Alternative Splicing Analysis in a Large-Scale Multiple Sclerosis Study
Source: Int J Mol Sci. 2024 Nov 7;25(22):11957. doi: 10.3390/ijms252211957 (PMC11593658; doi:10.3390/ijms252211957)

Table S1. Accession numbers of the sample-specific comparisons. AL: active lesion; CA: chronic active; NAWM: normal appearing white matter.

Sample ID	Lesion Type	Accession Number
S6	AL	SRR10248474
S6	AL	SRR10248477
S6	AL	SRR10248479
S6	NAWM	SRR10248426
S6	NAWM	SRR10248446
S6	NAWM	SRR10248448
S14	CA	SRR10248408
S14	CA	SRR10248409
S14	CA	SRR10248412
S14	CA	SRR10248440
S14	CA	SRR10248443
S14	NAWM	SRR10248423
S14	NAWM	SRR10248433
S14	NAWM	SRR10248449
S14	NAWM	SRR10248452
S9	CA	SRR10248415
S9	CA	SRR10248420
S9	CA	SRR10248439
S9	NAWM	SRR10248414
S9	NAWM	SRR10248430
S9	NAWM	SRR10248434
S9	NAWM	SRR10248444
S9	NAWM	SRR10248447

Table S2. Top 50 overexpressed genes in sample S6 AL vs NAWM

ENSEMBL	baseMean	log2FoldChange	P value	FDR	Gene Symbol
ENSG00000211598	61.40887189	3.358073346	9.15498E-11	1.67866E-06	*IGKV4-1*
ENSG00000206199	17.75290812	2.943291209	1.46939E-09	1.34714E-05	*ANKUB1*
ENSG00000039068	120.0125257	-1.797361702	4.15158E-08	0.000212318	*CDH1*
ENSG00000145703	254.8982805	1.962149708	4.64985E-08	0.000212318	*IQGAP2*
ENSG00000177575	1004.400271	1.898886006	5.78965E-08	0.000212318	*CD163*
ENSG00000105877	195.3092102	2.47680826	3.62111E-07	0.001106612	*DNAH11*
ENSG00000211934	38.80408951	2.578669624	4.76593E-07	0.001248402	*IGHV1-2*
ENSG00000118492	43.29878969	2.574905533	6.53957E-07	0.001498869	*ADGB*
ENSG00000162643	71.19196462	2.355560306	7.77243E-07	0.001583503	*DNAI3*
ENSG00000034239	48.54954016	2.398740277	1.40438E-06	0.002575066	*CLXN*
ENSG00000272398	379.1515392	2.367795278	1.74291E-06	0.00290527	*CD24*
ENSG00000133665	13.49126362	2.4403432	2.48329E-06	0.003794464	*DYDC2*
ENSG00000224373	59.90081173	2.239075798	3.029E-06	0.004272287	*IGHV4-59*
ENSG00000120262	125.5207639	1.468712179	4.4085E-06	0.005773881	*CCDC170*
ENSG00000260314	95.63672564	1.865432839	6.34938E-06	0.007761484	*MRC1*
ENSG00000065328	7.126236149	-2.298081247	8.58088E-06	0.007866949	*MCM10*
ENSG00000091181	15.62490037	2.307878752	8.28466E-06	0.007866949	*IL5RA*
ENSG00000121966	222.2816979	2.038563615	7.31338E-06	0.007866949	*CXCR4*
ENSG00000155761	139.5869347	2.249632431	7.35414E-06	0.007866949	*SPAG17*
ENSG00000265401	20.95017124	-2.315901992	7.83144E-06	0.007866949	
ENSG00000160401	39.1306099	2.213757402	1.65394E-05	0.014441233	*CFAP157*
ENSG00000164694	20.28898812	2.214878451	1.88391E-05	0.015701569	*FNDC1*
ENSG00000145423	153.4475006	1.928332026	2.3787E-05	0.018173263	*SFRP2*
ENSG00000197748	368.1429539	1.758839983	2.29827E-05	0.018173263	*CFAP43*
ENSG00000102174	77.1954467	1.666628837	3.64332E-05	0.019627304	*PHEX*
ENSG00000106483	81.36502251	1.820031104	3.78071E-05	0.019627304	*SFRP4*
ENSG00000111834	56.11493438	1.821465689	3.52117E-05	0.019627304	*RSPH4A*
ENSG00000112539	49.56403161	2.03722544	3.76505E-05	0.019627304	*C6orf118*
ENSG00000121207	209.6606966	1.734271623	3.58226E-05	0.019627304	*LRAT*
ENSG00000165309	132.3750071	1.739873545	3.47326E-05	0.019627304	*ARMC3*
ENSG00000165923	35.14925521	1.937189687	3.50647E-05	0.019627304	*AGBL2*
ENSG00000168658	73.5565266	1.915236614	3.85353E-05	0.019627304	*VWA3B*
ENSG00000168685	23.73384978	1.90445662	2.89403E-05	0.019627304	*IL7R*
ENSG00000188452	18.46208388	2.137244563	3.26284E-05	0.019627304	*CERKL*
ENSG00000211644	68.1204064	2.147884521	3.31446E-05	0.019627304	*IGLV1-51*
ENSG00000213085	72.44294647	2.130650678	3.18533E-05	0.019627304	*CFAP45*
ENSG00000158486	29.59926372	2.071210978	4.58878E-05	0.022740485	*DNAH3*
ENSG00000007174	332.7289818	1.824139823	5.08799E-05	0.023921384	*DNAH9*
ENSG00000163359	66.03983974	1.526685037	4.99738E-05	0.023921384	*COL6A3*
ENSG00000140795	58.38848635	2.087074728	5.55412E-05	0.02546008	*MYLK3*
ENSG00000132465	98.02114091	2.056679823	7.09261E-05	0.030244212	*JCHAIN*
ENSG00000163071	30.10427425	1.957539826	6.86995E-05	0.030244212	*SPATA18*
ENSG00000197826	17.95429019	2.056593967	7.08249E-05	0.030244212	*CFAP299*
ENSG00000082175	84.72871611	1.637327291	7.909E-05	0.03230815	*PGR*
ENSG00000153071	825.636917	1.105004773	7.92903E-05	0.03230815	*DAB2*
ENSG00000077327	72.8869733	1.876823902	8.61824E-05	0.034301366	*SPAG6*
ENSG00000138061	221.8922056	1.460920801	8.79234E-05	0.034301366	*CYP1B1*
ENSG00000133800	126.3202893	1.797290483	9.24253E-05	0.035306462	*LYVE1*
ENSG00000134028	19.09794775	1.918127559	9.69426E-05	0.03599465	*ADAMDEC1*
ENSG00000145623	456.3828503	1.266791274	9.81529E-05	0.03599465	*OSMR*

Table S3. Top 50 overexpressed genes in sample S9 CA vs NAWM

ENSEMBL	baseMean	log2FoldChange	p value	FDR	Gene Symbol
ENSG00000245067	75.4529339	2.49552572	1.3459E-08	0.000286957	*IGFBP7-AS1*
ENSG00000279690	59.3808041	2.54216831	2.2532E-08	0.000286957	
ENSG00000039537	29.191478	2.53304356	4.4555E-07	0.003782891	*C6*
ENSG00000106823	378.396797	1.78333132	2.5543E-06	0.016265411	*ECM2*
ENSG00000047457	3159.35988	1.76162406	5.3687E-06	0.027349159	*CP*
ENSG00000272398	379.151539	2.1292006	8.45E-06	0.035871659	*CD24*
ENSG00000019991	218.899378	1.6211616	1.05E-05	0.038205544	*HGF*
ENSG00000132465	98.0211409	2.16128719	2.4558E-05	0.078189431	*JCHAIN*
ENSG00000101916	70.3060999	1.60035967	3.4587E-05	0.088097017	*TLR8*
ENSG00000163885	48.0416741	1.90746442	3.3658E-05	0.088097017	*CFAP100*
ENSG00000198774	183.473235	1.81911382	3.9802E-05	0.092164378	*RASSF9*
ENSG00000165084	404.825742	1.46861666	6.9788E-05	0.125262588	*C8orf34*
ENSG00000171659	685.159672	1.53473049	7.3768E-05	0.125262588	*GPR34*
ENSG00000179813	26.8512811	2.0572326	6.508E-05	0.125262588	*FAM216B*
ENSG00000197747	573.33686	1.21219009	7.3612E-05	0.125262588	*S100A10*
ENSG00000164294	40.2708332	1.71335449	8.3114E-05	0.13231182	*GPX8*
ENSG00000155761	139.586935	1.90722347	9.4057E-05	0.140924895	*SPAG17*
ENSG00000105877	195.30921	1.78805	0.00013976	0.197766197	*DNAH11*
ENSG00000152936	94.529752	1.46055433	0.00014902	0.199776156	*LMNTD1*
ENSG00000132554	280.249723	1.58123877	0.00016644	0.201870249	*RGS22*
ENSG00000135046	667.308119	1.33696561	0.0001624	0.201870249	*ANXA1*
ENSG00000248801	53.9431494	1.83591562	0.00022309	0.258292626	*C8orf34-AS1*
ENSG00000116745	79.8162758	1.55862278	0.00023497	0.260208558	*RPE65*
ENSG00000226690	13.675516	1.8932191	0.00024523	0.260263452	
ENSG00000177575	1004.40027	1.18332513	0.00026127	0.266197158	*CD163*
ENSG00000188596	583.13856	1.46285267	0.00027892	0.273242354	*CFAP54*
ENSG00000006747	1107.0058	1.17989141	0.00029919	0.28224635	*SCIN*
ENSG00000173947	178.034076	1.3409565	0.00032454	0.295228543	*PIFO*
ENSG00000160838	7.58217851	1.85517445	0.00033689	0.295895033	*LRRC71*
ENSG00000154188	411.1145	1.26110254	0.00040363	0.332181734	*ANGPT1*
ENSG00000213085	72.4429465	1.7800244	0.00040429	0.332181734	*CFAP45*
ENSG00000162643	71.1919646	1.62116173	0.00044103	0.340404863	*DNAI3*
ENSG00000270550	16.9263375	1.79606708	0.00043157	0.340404863	*IGHV3-30*
ENSG00000162645	662.148052	1.22378097	0.00048533	0.363584066	*GBP2*
ENSG00000026025	9091.18189	1.18933157	0.00052779	0.37342937	*VIM*
ENSG00000147145	96.5266732	1.47668401	0.0005159	0.37342937	*LPAR4*
ENSG00000169385	47.4653304	1.39948759	0.00055438	0.381635953	*RNASE2*
ENSG00000140030	148.451651	1.40950832	0.00057138	0.382988571	*GPR65*
ENSG00000121316	89.4938444	1.37074309	0.00059566	0.389029666	*PLBD1*
ENSG00000175077	33.4541282	1.48679856	0.00062766	0.399679032	*RTP1*
ENSG00000205835	80.1600578	1.61827179	0.00066246	0.411550536	*GMNC*
ENSG00000118492	43.2987897	1.73196732	0.00073199	0.43724422	*ADGB*
ENSG00000144354	29.7283792	1.4843744	0.00073815	0.43724422	*CDCA7*
ENSG00000092969	911.26171	1.09600535	0.00103678	0.586838719	*TGFB2*
ENSG00000132965	335.138721	1.22315201	0.00103042	0.586838719	*ALOX5AP*
ENSG00000110077	605.814433	0.93687913	0.00111428	0.616996806	*MS4A6A*
ENSG00000136918	11.637692	1.67589829	0.0011729	0.635638922	*WDR38*
ENSG00000034239	48.5495402	1.53174235	0.0016218	0.649329941	*CLXN*
ENSG00000091181	15.6249004	1.65460838	0.0013926	0.649329941	*IL5RA*
ENSG00000141232	1300.53716	1.0242855	0.00159234	0.649329941	*TOB1*

Table S4. Top 50 overexpressed in sample S14 CA vs NAWM

ENSEMBL	baseMean	log2FoldChange	P value	FDR	Gene Symbol
ENSG00000211592	1784.875869	3.998796543	6.64876E-22	1.18188E-17	*IGKC*
ENSG00000211938	173.3356138	3.89399377	5.25274E-20	4.66864E-16	*IGHV3-7*
ENSG00000163885	89.6152127	3.167514495	2.14961E-17	1.27371E-13	*CFAP100*
ENSG00000272398	580.5446218	3.377801002	5.92424E-17	2.63273E-13	*CD24*
ENSG00000124491	881.320999	2.515192517	7.92173E-16	2.81633E-12	*F13A1*
ENSG00000205835	110.0274908	3.032773349	1.03881E-15	3.07766E-12	*GMNC*
ENSG00000132465	183.2699715	3.374014288	3.00556E-15	6.67835E-12	*JCHAIN*
ENSG00000165457	90.00210278	2.680035629	2.86533E-15	6.67835E-12	*FOLR2*
ENSG00000211896	3116.741877	3.285655274	3.52127E-15	6.95491E-12	*IGHG1*
ENSG00000153347	73.40745085	3.297121024	1.08676E-14	1.93182E-11	*FAM81B*
ENSG00000198774	221.0552752	2.625866715	1.3368E-13	2.16027E-10	*RASSF9*
ENSG00000157330	39.56511708	3.187624424	1.62883E-13	2.41284E-10	*CFAP107*
ENSG00000162598	79.23552444	3.070921959	2.10635E-13	2.88018E-10	*C1orf87*
ENSG00000155659	435.3265925	1.96805898	2.34342E-13	2.97547E-10	*VSIG4*
ENSG00000105877	313.0952069	2.897467556	3.13872E-13	3.59889E-10	*DNAH11*
ENSG00000176601	88.19437585	3.273236839	3.23933E-13	3.59889E-10	*MAP3K19*
ENSG00000162643	108.5616334	2.918555149	5.87054E-13	6.13851E-10	*DNAI3*
ENSG00000110077	885.0564178	1.572085188	7.37255E-13	7.2808E-10	*MS4A6A*
ENSG00000039139	276.3754475	2.536402591	9.06774E-13	8.0594E-10	*DNAH5*
ENSG00000110079	73.03926301	2.266522134	8.67094E-13	8.0594E-10	*MS4A4A*
ENSG00000131951	203.4590144	2.627258226	1.40901E-12	1.19269E-09	*LRRC9*
ENSG00000180638	79.11539049	2.750475892	2.00212E-12	1.61772E-09	*SLC47A2*
ENSG00000211959	33.5856733	3.17511463	4.45271E-12	3.44136E-09	*IGHV4-39*
ENSG00000120708	268.3937247	1.959618543	4.72194E-12	3.49738E-09	*TGFBI*
ENSG00000179813	47.94559569	3.16872989	9.36968E-12	6.66222E-09	*FAM216B*
ENSG00000034239	109.5636494	2.592674302	1.68292E-11	1.1506E-08	*CLXN*
ENSG00000019991	307.154295	2.116615888	1.92879E-11	1.26986E-08	*HGF*
ENSG00000155761	212.6678428	2.915539573	2.72353E-11	1.72906E-08	*SPAG17*
ENSG00000177575	1216.597264	2.05085923	4.00058E-11	2.45221E-08	*CD163*
ENSG00000179178	138.4184481	-2.195741031	5.51482E-11	3.26771E-08	*TMEM125*
ENSG00000213085	87.44363184	2.794111802	5.96388E-11	3.4198E-08	*CFAP45*
ENSG00000092969	1219.196722	1.600597204	1.05876E-10	5.88142E-08	*TGFB2*
ENSG00000133800	362.6537023	2.024821442	1.20388E-10	6.48489E-08	*LYVE1*
ENSG00000135046	653.9823156	2.047012651	1.38404E-10	7.23607E-08	*ANXA1*
ENSG00000162493	550.8112957	1.577154422	1.7193E-10	8.73207E-08	*PDPN*
ENSG00000165168	1050.919839	1.774258895	3.42462E-10	1.691E-07	*CYBB*
ENSG00000102383	208.5823367	1.611675358	4.12794E-10	1.98319E-07	*ZDHHC15*
ENSG00000118492	63.67055029	2.834140465	7.88765E-10	3.67516E-07	*ADGB*
ENSG00000211897	31.34432628	2.655627485	8.06318E-10	3.67516E-07	*IGHG3*
ENSG00000144354	48.0740965	2.172868389	1.0314E-09	4.47176E-07	*CDCA7*
ENSG00000182329	120.8804553	2.359375984	1.01781E-09	4.47176E-07	*KIAA2012*
ENSG00000188931	65.82734115	2.686985942	1.19186E-09	5.04439E-07	*CFAP126*
ENSG00000260314	142.6738709	1.889814309	1.97715E-09	8.17346E-07	*MRC1*
ENSG00000152611	22.29570208	2.599267693	2.6216E-09	1.05912E-06	*CAPSL*
ENSG00000147145	140.1420192	1.878985241	2.792E-09	1.1029E-06	*LPAR4*
ENSG00000106823	383.8262812	2.003490716	2.99013E-09	1.15246E-06	*ECM2*
ENSG00000166596	101.1242328	2.609133372	3.04711E-09	1.15246E-06	*CFAP52*
ENSG00000189184	476.5560756	1.686477887	3.32935E-09	1.23297E-06	*PCDH18*
ENSG00000197748	459.0950423	2.074632538	3.7792E-09	1.371E-06	*CFAP43*
ENSG00000143297	16.11791675	2.623139754	4.26438E-09	1.51607E-06	*FCRL5*

Table S5. GSEA analysis of differentially expressed genes in AL vs NAWM S6

**S6_ALvsNAWM_GOBP_pos** **G1S DETAILS**	**ES**	**NES**	**NOM p-val**	**FDR q-val**	**FWER p-val**	**RANK AT MAX**
GOBP_CILIUM_MOVEMENT	137	0.72	3.04	0	0	0
GOBP_AXONEME_ASSEMBLY	78	0.76	2.93	0	0	0
GOBP_CILIUM_OR_FLAGELLUM_DEPENDENT_CELL_MOTILITY	104	0.7	2.78	0	0	0
GOBP_MICROTUBULE_BUNDLE_FORMATION	106	0.68	2.73	0	0	0
GOBP_AXONEMAL_DYNEIN_COMPLEX_ASSEMBLY	34	0.82	2.71	0	0	0
GOBP_SPERM_MOTILITY	84	0.68	2.7	0	0	0
GOBP_EXTRACELLULAR_TRANSPORT	42	0.75	2.64	0	0	0
GOBP_REGULATION_OF_CILIUM_MOVEMENT	24	0.82	2.46	0	0	0
GOBP_MOTILE_CILIUM_ASSEMBLY	51	0.68	2.39	0	0	0
GOBP_REGULATION_OF_MICROTUBULE_BASED_MOVEMENT	38	0.68	2.32	0	0	0
GOBP_OUTER_DYNEIN_ARM_ASSEMBLY	20	0.81	2.31	0	0	0
GOBP_CILIUM_ORGANIZATION	382	0.5	2.31	0	0	0
GOBP_REGULATION_OF_CILIUM_BEAT_FREQUENCY	15	0.84	2.23	0	0	0.002
GOBP_MICROTUBULE_BASED_MOVEMENT	354	0.47	2.21	0	0	0.003
GOBP_POSITIVE_REGULATION_OF_OSTEOBLAST_DIFFERENTIATION	64	0.59	2.19	0	0.001	0.009
GOBP_INNER_DYNEIN_ARM_ASSEMBLY	15	0.85	2.19	0	0.001	0.009
GOBP_ZYMOGEN_ACTIVATION	52	0.57	2.08	0	0.003	0.047
GOBP_SPERM_FLAGELLUM_ASSEMBLY	30	0.66	2.07	0	0.004	0.059
GOBP_CELLULAR_RESPONSE_TO_INTERLEUKIN_1	72	0.55	2.06	0	0.005	0.084
S6_ALvsNAWM_GOBP_neg						
GOBP_REGULATION_OF_POSTSYNAPTIC_MEMBRANE_POTENTIAL	122	-0.5	-2.45	0	0.001	0.001
GOBP_POSTSYNAPSE_ASSEMBLY	40	-0.7	-2.42	0	0	0.001
GOBP_POSITIVE_REGULATION_OF_EXCITATORY_POSTSYNAPTIC_POTENTIAL	31	-0.7	-2.36	0	0	0.001
GOBP_POSTSYNAPTIC_SPECIALIZATION_ASSEMBLY	28	-0.7	-2.35	0	0	0.001
GOBP_MODULATION_OF_EXCITATORY_POSTSYNAPTIC_POTENTIAL	44	-0.6	-2.33	0	0	0.001
GOBP_CHEMICAL_SYNAPTIC_TRANSMISSION_POSTSYNAPTIC	106	-0.5	-2.3	0	0	0.003
GOBP_POSTSYNAPTIC_SPECIALIZATION_ORGANIZATION	43	-0.6	-2.26	0	0.001	0.012
GOBP_GLUTAMATE_RECEPTOR_SIGNALING_PATHWAY	48	-0.6	-2.24	0	0.001	0.014
GOBP_LIGAND_GATED_ION_CHANNEL_SIGNALING_PATHWAY	29	-0.7	-2.24	0	0.001	0.015
GOBP_VESICLE_MEDIATED_TRANSPORT_IN_SYNAPSE	209	-0.5	-2.23	0	0.001	0.015
GOBP_CALCIUM_ION_REGULATED_EXOCYTOSIS	60	-0.5	-2.21	0	0.002	0.022
GOBP_REGULATION_OF_TRANS_SYNAPTIC_SIGNALING	426	-0.4	-2.19	0	0.002	0.024
GOBP_EXCITATORY_SYNAPSE_ASSEMBLY	33	-0.6	-2.19	0	0.002	0.026
GOBP_NEGATIVE_REGULATION_OF_AXON_EXTENSION_INVOLVED_IN_AXON_GUIDANCE	26	-0.7	-2.18	0	0.002	0.027
GOBP_SYNAPTIC_VESICLE_EXOCYTOSIS	93	-0.5	-2.16	0	0.002	0.033
GOBP_CALCIUM_ION_REGULATED_EXOCYTOSIS_OF_NEUROTRANSMITTER	20	-0.7	-2.16	0	0.002	0.038
GOBP_REGULATION_OF_NEUROTRANSMITTER_RECEPTOR_ACTIVITY	56	-0.5	-2.15	0	0.002	0.041
GOBP_PROTEIN_LOCALIZATION_TO_SYNAPSE	75	-0.5	-2.14	0	0.002	0.051
GOBP_POSTSYNAPTIC_DENSITY_ASSEMBLY	22	-0.7	-2.13	0	0.003	0.06
GOBP_SYNAPTIC_VESICLE_RECYCLING	79	-0.5	-2.11	0	0.003	0.074

Table S6. GSEA analysis of differentially expressed genes in CA vs NAWM S9

**S9_CAvsNAWM_GOBP_pos**<br>**GS DETAILS**	**ES**	**NES**	**NOM p-val**	**FDR q-val**	**FWER p-val**	**RANK AT MAX**
GOBP_B_CELL_MEDIATED_IMMUNITY	156	0.65	2.99	0	0	0
GOBP_AXONEME_ASSEMBLY	91	0.69	2.94	0	0	0
GOBP_ADAPTIVE_IMMUNE_RESPONSE	498	0.55	2.91	0	0	0
GOBP_LYMPHOCYTE_MEDIATED_IMMUNITY	295	0.56	2.88	0	0	0
GOBP_ADAPTIVE_IMMUNE_RESPONSE_BASED_ON_SOMATIC_RECOMBINATION_OF_IMMUNE_RECEPTORS_BUILT_FROM_IMMUNOGLOBULIN_SUPERFAMILY_DOMAINS	311	0.56	2.86	0	0	0
GOBP_CILIUM_MOVEMENT	175	0.59	2.77	0	0	0
GOBP_HUMORAL_IMMUNE_RESPONSE_MEDIATED_BY CIRCULATING_IMMUNOGLOBULIN	45	0.73	2.69	0	0	0
GOBP_COMPLEMENT_ACTIVATION	52	0.7	2.66	0	0	0
GOBP_LEUKOCYTE_MEDIATED_IMMUNITY	384	0.52	2.66	0	0	0
GOBP_AXONEMAL_DYNEIN_COMPLEX_ASSEMBLY	38	0.73	2.57	0	0	0
GOBP_COMPLEMENT_ACTIVATION_CLASSICAL_PATHWAY	33	0.75	2.57	0	0	0
GOBP_MICROTUBULE_BUNDLE_FORMATION	120	0.58	2.56	0	0	0
GOBP_EXTRACELLULAR_TRANSPORT	46	0.69	2.56	0	0	0
GOBP_PEPTIDE_ANTIGEN_ASSEMBLY_WITH_MHC_PROTEIN_COMPLEX	18	0.86	2.56	0	0	0
GOBP_MONOCYTE_CHEMOTAXIS	50	0.66	2.54	0	0	0
GOBP_CILIUM_OR_FLAGELLUM_DEPENDENT_CELL_MOTILITY	136	0.56	2.53	0	0	0
GOBP_HUMORAL_IMMUNE_RESPONSE						
GOBP_CELL_KILLING	160	0.53	2.49	0	0	0
GOBP_POSITIVE_REGULATION_OF_CHEMOKINE_PRODUCTION	62	0.62	2.46	0	0	0
GOBP_ANTIGEN_PROCESSING_AND_PRESENTATION OF_PEPTIDE_ANTIGEN	68	0.61	2.46	0	0	0
S9_CAvsNAWM_GOBP_neg						
GOBP_SHORT_CHAIN_FATTY_ACID_METABOLIC_PROCESS	15	-0.72	-2.02	0	0.134	0.161
GOBP_FATTY_ACID_BETA_OXIDATION	74	-0.48	-1.94	0	0.201	0.391
GOBP_OXIDATIVE_PHOSPHORYLATION	130	-0.43	-1.92	0	0.178	0.483
GOBP_MITOCHONDRIAL_ELECTRON_TRANSPORT_CYTOCHROME_C_TO_OXYGEN	20	-0.64	-1.92	0	0.137	0.492
GOBP_FATTY_ACID_CATABOLIC_PROCESS	102	-0.45	-1.9	0	0.152	0.61
GOBP_ATP_SYNTHESIS_COUPLED_ELECTRON_TRANSPORT	88	-0.46	-1.89	0	0.153	0.682
GOBP_AEROBIC_RESPIRATION	177	-0.41	-1.88	0	0.151	0.735
GOBP_MONOCARBOXYLIC_ACID_CATABOLIC_PROCESS	123	-0.42	-1.86	0	0.152	0.79
GOBP_2_OXOGLUTARATE_METABOLIC_PROCESS	15	-0.66	-1.85	0	0.158	0.842
GOBP_CHEMICAL_SYNAPTIC_TRANSMISSION_POSTSYNAPTIC	104	-0.43	-1.85	0	0.145	0.85
GOBP_INHIBITORY_POSTSYNAPTIC_POTENTIAL	17	-0.63	-1.85	0.008	0.136	0.855
GOBP_REGULATION_OF_PROTON_TRANSPORT	16	-0.66	-1.84	0.004	0.135	0.874
GOBP_REGULATION_OF_TRANSCRIPTION_OF_NUCLEOLAR_LARGE_RRNA_BY_RNA_POLYMERASE_I	15	-0.66	-1.82	0	0.152	0.922

Table S7. GSEA analysis of differentially expressed genes in CA vs NAWM S14

**S14_CAvsNAWM_GOBP_pos**<br>**GS DETAILS**	**ES**	**NES**	**NOM p-val**	**FDR q-val**	**FWER p-val**	**RANK AT MAX**
GOBP_AXONEME_ASSEMBLY	85	0.79	2.88	0	0	0
GOBP_CILIUM_MOVEMENT	149	0.68	2.74	0	0	0
GOBP_MICROTUBULE_BUNDLE_FORMATION	112	0.7	2.73	0	0	0
GOBP_AXONEMAL_DYNEIN_COMPLEX_ASSEMBLY	37	0.83	2.65	0	0	0
GOBP_B_CELL_MEDIATED_IMMUNITY	113	0.68	2.63	0	0	0
GOBP_EXTRACELLULAR_TRANSPORT	45	0.77	2.54	0	0	0
GOBP_ADAPTIVE_IMMUNE_RESPONSE_BASED_ON_SOMATIC_ RECOMBINATION_OF_IMMUNE_RECEPTORS_BUILT_FROM_ IMMUNOGLOBULIN_SUPERFAMILY_DOMAINS	245	0.6	2.54	0	0	0
GOBP_CILIUM_OR_FLAGELLUM_DEPENDENT_CELL_MOTILITY	111	0.65	2.53	0	0	0
GOBP_HUMORAL_IMMUNE_RESPONSE_MEDIATED_BY_CIRCULATING_IMMUNOGLOBULIN	38	0.78	2.52	0	0	0
GOBP_MOTILE_CILIUM_ASSEMBLY	53	0.71	2.47	0	0	0
GOBP_ADAPTIVE_IMMUNE_RESPONSE	349	0.56	2.45	0	0	0
GOBP_LYMPHOCYTE_MEDIATED_IMMUNITY	220	0.58	2.43	0	0	0
GOBP_COMPLEMENT_ACTIVATION	42	0.74	2.42	0	0	0
GOBP_REGULATION_OF_CILIUM_MOVEMENT	25	0.81	2.38	0	0	0
GOBP_COMPLEMENT_ACTIVATION_CLASSICAL_PATHWAY	30	0.78	2.38	0	0	0
GOBP_LEUKOCYTE_MEDIATED_IMMUNITY	294	0.54	2.33	0	0	0
GOBP_CILIUM_ORGANIZATION	393	0.53	2.32	0	0	0
GOBP_HUMORAL_IMMUNE_RESPONSE	114	0.6	2.3	0	0	0
GOBP_SPERM_MOTILITY	86	0.63	2.29	0	0	0
GOBP_POSITIVE_REGULATION_OF_OSTEOBLAST_DIFFERENTIATION	59	0.66	2.29	0	0	0
S14_CAvsNAWM_GOBP_neg						
GOBP_ACETYL_COA_METABOLIC_PROCESS	29	-0.6	-2.07	0	0.101	0.117
GOBP_OLIGODENDROCYTE_DEVELOPMENT	46	-0.5	-2.05	0	0.065	0.146
GOBP_GPI_ANCHOR_METABOLIC_PROCESS	30	-0.6	-2.04	0	0.047	0.158
GOBP_ENSHEATHMENT_OF_NEURONS	135	-0.4	-1.98	0	0.079	0.315
GOBP_AXON_ENSHEATHMENT_IN_CENTRAL_NERVOUS_SYSTEM	25	-0.6	-1.95	0	0.102	0.468
GOBP_STEROL_METABOLIC_PROCESS	119	-0.4	-1.93	0	0.106	0.543
GOBP_STEROL_BIOSYNTHETIC_PROCESS	56	-0.5	-1.93	0	0.091	0.544
GOBP_OLIGODENDROCYTE_DIFFERENTIATION	97	-0.4	-1.88	0	0.132	0.723
GOBP_RESPONSE_TO_IMMOBILIZATION_STRESS	16	-0.6	-1.8	0.011	0.259	0.942
GOBP_THIOESTER_METABOLIC_PROCESS	77	-0.4	-1.79	0	0.245	0.951
GOBP_REGULATION_OF_CAMP_MEDIATED_SIGNALING	20	-0.6	-1.74	0	0.362	0.992
GOBP_TAIL_ANCHORED_MEMBRANE_PROTEIN_INSERTION_INTO_ER_MEMBRANE	17	-0.6	-1.72	0.005	0.392	0.997
GOBP_N_ACETYLGLUCOSAMINE_METABOLIC_PROCESS	17	-0.6	-1.71	0.018	0.419	0.999
GOBP_RRNA_METHYLATION	27	-0.5	-1.68	0.011	0.498	1
GOBP_THIOESTER_BIOSYNTHETIC_PROCESS	41	-0.4	-1.68	0.006	0.474	1
GOBP_SENSORY_PERCEPTION_OF_BITTER_TASTE	21	-0.5	-1.67	0.006	0.475	1
GOBP_DETECTION_OF_CHEMICAL_STIMULUS_INVOLVED _IN_SENSORY_PERCEPTION_OF_TASTE	20	-0.5	-1.66	0.013	0.463	1
GOBP_TRNA_PROCESSING	133	-0.4	-1.66	0	0.444	1
GOBP_NUCLEOSIDE_BISPHOSPHATE_BIOSYNTHETIC_PROCESS	52	-0.4	-1.66	0.003	0.431	1
GOBP_NEGATIVE_REGULATION_OF_TOR_SIGNALING	68	-0.4	-1.64	0.007	0.492	1

Figure S1. Representative graphs from rMAPS2 analysis showing different splicing mechanisms and RNA binding motifs that are significantly involved in differential splicing of MS and non-MS tissues. (**A**) Skipped Exon (SE), (**B**) Mutually Exclusive Exons (MXE), (**C**) Retained Intron (RI), (**D**) Alternative 3′ Splice Site (A3SS), (**E**) Alternative 5′ Splice Site (A5SS) events. Enriched RBP motifs are highlighted.

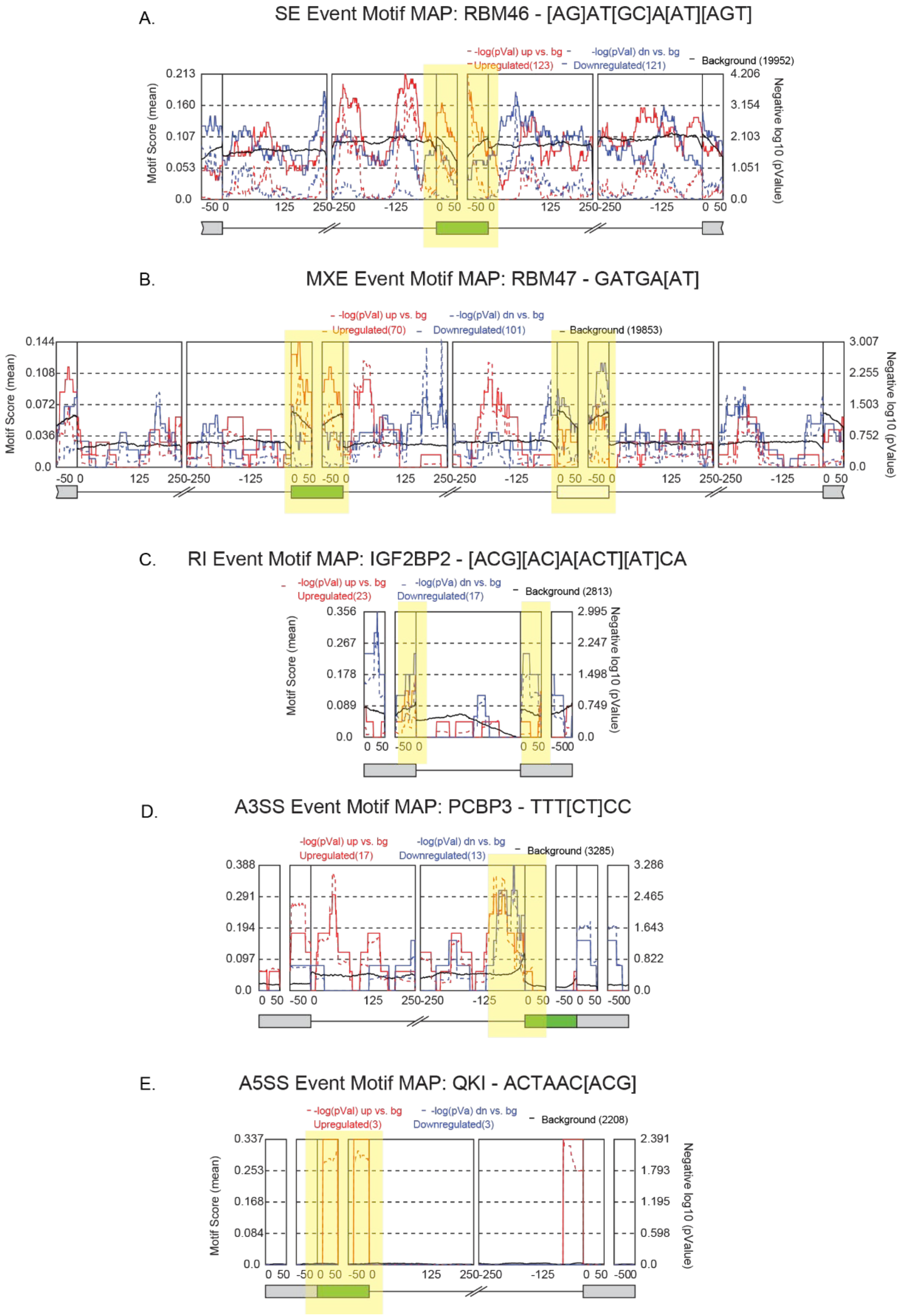

Figure S2. Gene ontology biological processes enrichment graphs of differentially spliced genes in (**A**) AL compared to NAWM from donor S6 and (**B**) CA compared to NAWM from donors S9 and S14.

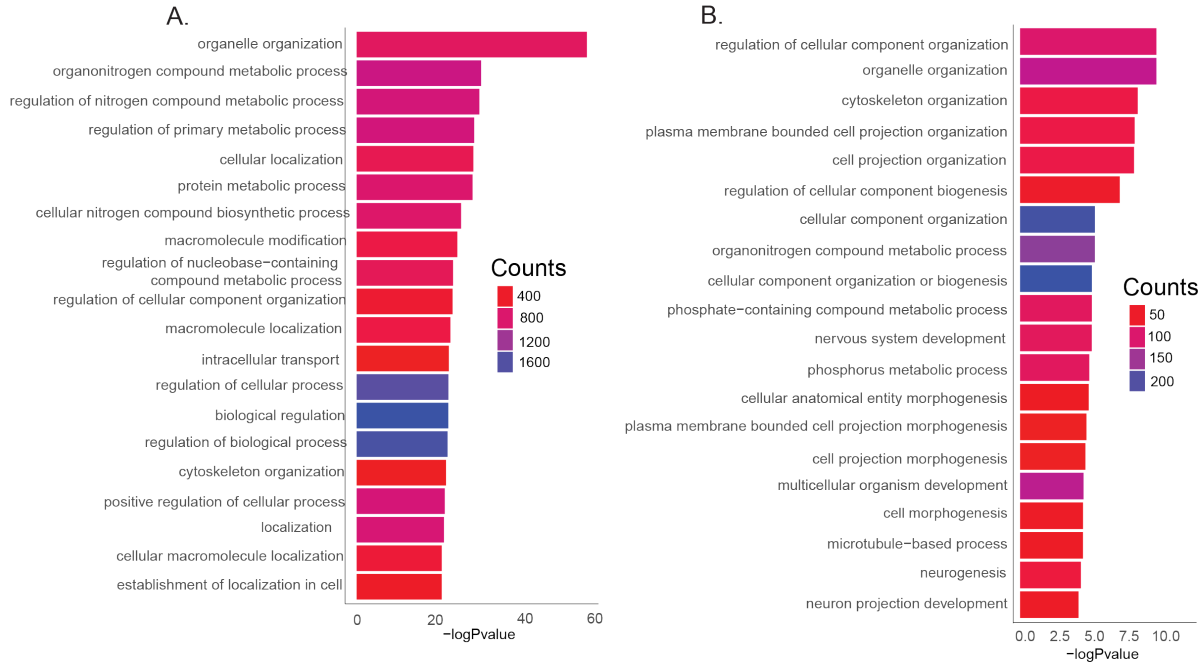

Supplement: Supplementary file 1 [file ijms-25-11957-s001.zip › ijms-3266072-supplementary.pdf]
